# Textural, Sensory and Volatile Compounds Analyses in Formulations of Sausages Analogue Elaborated with Edible Mushrooms and Soy Protein Isolate as Meat Substitute

**DOI:** 10.3390/foods11010052

**Published:** 2021-12-27

**Authors:** Xinyue Yuan, Wei Jiang, Dianwei Zhang, Huilin Liu, Baoguo Sun

**Affiliations:** Beijing Advanced Innovation Center for Food Nutrition and Human Health, Beijing Engineering and Technology Research Center of Food Additives, Beijing Technology and Business University, 11 Fucheng Road, Beijing 100048, China; amy_yuan0801@163.com (X.Y.); 18811025200@163.com (W.J.); zhangdianwei@btbu.edu.cn (D.Z.); sunbg@btbu.edu.cn (B.S.)

**Keywords:** meat analogue, mushroom, sausages, GC-MS, flavor, texture

## Abstract

In this study, edible mushroom and soybean protein isolate (SPI) were used to prepare a fibrous meat analogue using thermos-extrusion and the extruded mushroom-based meat analogue as meat replacer was further developed with different formulations in fabricating sausage analogues. The effect of water content (35%, 70% and 100%), three types of edible mushroom (*Lentinus edodes*, *Pleurotus ostreatus*, *Coprinus comatus* and a mixture of equal proportions) and their amounts (from 15% to 100%) on the physicochemical and structural profiles were studied. The results showed that the extruded mushroom-based meat analogue prepared from *Coprinus comatus* (15% addition) and SPI with a water content of 35% exhibited close textural profiles to real beef. Furthermore, a texture profile analysis (TPA) combined with a principal component analysis (PCA) was conducted to compare and assess the textural traits of the sausage analogues with similar commercial products. The characterization and comparison of the flavor profile of post-processing mushroom-based meat sausage analogues (MMSA) were performed using headspace-phase microextraction (HS-SPME), coupled with gas chromatography-mass spectrometry (GC-MS). A total of 64 volatile compounds were identified, and the content in dried-processing treatment was significantly higher than for steamed-processing, which indicated that the natural fermentation process contributed to the increase in aroma substances in the non-animal sourced sausage. This study developed a feasible method to fabricate a meat replacement and to create high added-value products, which offer an opportunity for developing non-animal products with satisfactory sensory properties and flavor profiles.

## 1. Introduction

The United Nations Department of Economic and Social Affairs has estimated that by 2050 the world population will reach 9.7 billion, and the demand gap in the global meat market will reach 38 million tons [1]. This gap cannot be satisfied through a reliance on traditional methods of animal breeding and processing to produce meat products. Increasing the production and consumption of meat products also leads to concerns about water and land requirements, pollution and CO_2_ emissions and biodiversity loss [2]. There is also strong evidence of specific adverse effects, the increased risks of colorectal cancer and cardiovascular disease associated with high intakes of processed meat [3]. The development of “artificial meat” has been proposed as a solution to this problem and to combat the adverse effects and demand gap related to meat [4]. A healthy, green, highly efficient and sustainable food in the form of “artificial meat” could promote the transformation and upgrading of global meat consumption. This would not only accelerate the development of traditional meat in the direction of deep processing and high added value, but also attract many authoritative organizations at home and abroad to develop “artificial meat” products. In fact, there are two kinds of artificial meat, one of which is lab-grown meat generated from cell cultures as opposed to whole organisms or animals, and the other is imitation meat, made from vegetable protein or other ingredients to mimic the flavor and taste of meat. However, the relatively poor texture, flavor, juiciness, chewiness and appearance of meat analogues currently available remain hurdles to overcome.

Most notably, Shurtleff et al. [5] provided a timetable detailing events in the history of developing meat substitutes between 965 CE and 2014, when a soy-based tofu product was made in China. Thousands of years ago, the primary protein ingredients used in traditional meat analogues were seitan, tofu, yuba, tempeh et al. However, as Kyriakopoulou et al. [6] summarized, the sources of the plant-based proteins most widely used in meat analogues in recent years are soy and the wheat protein gluten, while other proteins have also been used in meat analogues, including legumes from pea, faba bean, kidney bean, and others, and fungi from mycoprotein and mushrooms. In many cultures, mushrooms, which are well-known macro-fungi with distinctive hypogeous or epigeous fruiting bodies, are a delicacy highly prized for their particular aromas and textures [7]. Edible mushrooms are considered to be highly nutritious foods, with the principal nutritional compounds of mushrooms being polysaccharides. Mushrooms contain essential amino acids and polyunsaturated fatty acids, which are the only non-animal source of vitamin D, and contain a large number of B vitamins and various minerals required for human physiological functions [8,9]. Moreover, mushrooms also contain several bioactive components such as polysaccharides, proteins, peptides, proteoglycans, phenolic compounds, terpenes and lectins [7].

Research on plant-based meat has steadily increased since 2010, indicating that interest in plant-based meat analogues has been growing. Many studies have reported the use of mushrooms in meat products because of their unique flavors and health-promoting properties, while few have focused on use of mushrooms in meat analogues. Introducing mushrooms might also play an important role in meat analogues-based products by providing nutrients, and promoting the development of chief organoleptic properties, such as the appearance, texture and flavor of the product.

Thus, this study aims to manufacture a mushroom-based meat analogue and to produce value-added products that could meet the demand by consumer groups. In the present study, the effect of edible mushrooms (both species and the ratio with SPI) on meat analogues was evaluated in terms of their physicochemical traits and textural properties. Moreover, the mushroom-based meat analogue prepared as a meat replacement was investigated in sausage production with different formulations and processing. Textural profiles, physicochemical and sensory analyses were conducted to investigate and compare with the commercial sausage products. Furthermore, to analyze the flavor profile, the volatile compounds from steamed and dried MMSA are identified using headspace solid-phase microextraction (HS-SPME) combined with gas chromatography-mass spectrometry (GC-MS).

## 2. Materials and Methods

### 2.1. Materials

Three edible mushroom species (*Lentinus edodes*, *Coprinus comatus* and *Pleurotus ostreatus*) were purchased from Guanfa Food Co., Ltd. (Fujian, China). The soybean protein isolate (protein ≥ 90% on a dry weight basis) was provided by Linyi Shansong Biological Products Co., Ltd. (Shandong, China). Food grade additives and beef were obtained from a local supermarket. Isoamyl phenylacetate as the internal standard was purchased from TCI Co., Ltd. (purity ≥ 98.0%, Shanghai, China).

### 2.2. Preparation of Edible Mushroom-Based Meat Analogue

A commercial blender was purchased from Joyoung Co., Ltd. (Jinan, China) and used to grind the edible mushroom to powder and then sieved with a screen of 60-mesh. To ensure uniformity, the integration of mushroom powder (from 15% to 100%) and SPI and distilled water (35%, 70% and 100%, *w*/*w*) was carried out by the blender. The meat analogue was extruded using a BP-8176-AT single-screw extruder (Baopin Precision Instrument Co., Ltd., Dongguan, China). The speed of the single-lead screw with a diameter of 20 mm was set to 45 rpm and the temperatures from the first to third zones were set at 80, 140 and 80 °C, respectively. The input of the mushroom and SPI mixture was fixed at a constant speed. The extrudate was collected immediately as it exited the die then stored at −18 °C after cooling to room temperature.

### 2.3. Preparation of Mushroom-Based Meat Sausage Analogue

The extruded fibrous meat analogue was used as the main ingredient for producing sausages. After soaking in distilled water for 8 h, meat analogue was minced and mixed with corn starch (5%), konjac powder (10%), salt (3%), sugar (5%), pepper (0.2%), sodium glutamate (0.16%), carrageenan (0.3%), five-spice powder (2%) and sodium erythorbate (0.4%). The key factorial design (4 factors at 3 levels) consisted of 9 different formulations (Table 1), based on three independent variables, including egg white powder, X1 (3%, 4% and 5%); meat flavor powder, X2 (0.1%, 0.2% and 0.3%); oil, X3 (17%, 19% and 21%); and red yeast rice, X4 (0.01%, 0.02% and 0.03%). The mixture was homogenized then filled into cellulose casings.

### 2.4. Microstructural, Physicochemical and Textural Properties

The imaging of the interior microstructure of the meat analogues was obtained from a scanning electron microscope (SEM, SU 8020, Hitachi, Tokyo, Japan). The meat analogues were cut into small pieces (5 mm × 8 mm × 0.8 mm) and immersed in glutaraldehyde (2.5%, *v*/*v*) at 25 °C for 24 h. The sample was then rinsed with distilled water and dehydrated in a series of graded ethanol solutions then dried out. Finally, after coating with gold, the sample was observed using SEM.

A Testo205 pH sensor (Testo AG, Lenzkirch, Germany) was used to measure the pH value, and the water activity (*a_w_*) was measured using a benchtop meter (AquaLab 4TE, Decagon Devices, Pullman, WA, USA). The color of each sausage analogue was determined using a colorimeter (CM-600d1, Konica Minolta Inc., Tokyo, Japan). The chromaticity parameters L* (lightness), a* (redness), and b* (yellowness) were measured and compared. The instrument was calibrated with standard white and black reference tiles before analysis [10]. Cook yield was determined by measuring the weight of each sausage analogue before and after cooking. The values were calculated by Wong et.al as follows [11]:

(1) $$\mathrm{Cook}\;\mathrm{yield}\;\left(\%\right)=\frac{\mathrm{Cooked}\;\mathrm{weight}}{\mathrm{Pre}-\mathrm{cooked}\;\mathrm{weitght}}\times 100\;$$

The results are expressed as a percentage. The texture profiles of the different meat analogue and sausage analogue samples were analyzed using a CT3 texture analyzer (Brookfield, Middleboro, MA, USA). The hardness, springiness, fracture property and viscous force of the sausage analogue were measured using two cycles of compression with the accompanying probe attachment (TA44, cylinder diameter = 4 mm). The test was performed for up to 50% compression and the speed was set at 2.00 mm/s. For selecting an optimal mushroom variety and the proportion of mushroom with SPI to mimic real meat, both *a_w_* and texture profiles of meat analogues were compared with beef.

### 2.5. Sensory Analysis of Mushroom-Based Meat Sausage Analogue

The sensory evaluation focused on color, mouthfeel, flavor, texture and overall acceptability. To mimic the consumers’ evaluation more objectively and actually, 32 untrained panelists (consisting of 16 females and 16 males, age 20–30 years) were recruited from the students and staff of BTBU University randomly. The sausage analogues were cooked at 180 °C for 10 min using an electric steamer (ZN28YK807-150, Supor Co., Ltd., Zhejiang, China) and brought to room temperature before assessment. The sausage analogue samples were cut into slices with a thickness of 5 mm then distributed to panelists. Water was provided for panelists to rinse their mouths between the test samples and coffee bean was provided to neutralize the odor after each sample. The scoring of each sample was performed according to a 9-point hedonic scale to assess consumers’ preferences of each attribute [12]. On the 9-point hedonic scale, ‘9′ corresponded to ‘strongly like and ‘1′ corresponded to ‘strongly dislike’. Specifically, sensory characteristics were: color (9 = light and rosy, 1 = dark and brown), mouthfeel (9 = juicy and elastic, 1 = dry and hard), flavor (9 = adorable sausage flavor, 1 = no sausage flavor), textual (9 = tightness and even, 1 = loose and uneven), and overall impression (9 = extremely good, 1 = extremely bad) through a line scale. All assessments were determined in the sensory evaluation laboratory, equipped with partitioned cabinets and white light [13,14,15]. Besides, to avoid the impact of shocks, all panelists were informed in advance that the samples were a novel product designed to replace the conventional animal-meat sausages.

### 2.6. Volatile Compounds

Extraction of volatile compounds was carried out using HS-SPME. An isoamyl phenylacetate internal standard solution (100 µL of a 50 µg mL^−1^ solution) and a sausage analogue sample (5 g) were introduced into a 20-mL headspace vial sealed with a Teflon/silicone septum. The temperature of the container was then controlled thermostatically at 60 °C for 20 min, and an SPME fiber of 50/30 mm divinylbenzene/carboxen/polydimethylsiloxane (DVB/CAR/PDMS, Supelco, Bellefonte, PA, USA) was introduced to extract the volatile compounds for about 30 min.

The volatile substances were then analyzed by a GC-MS (Agilent 7890A-5975C, Agilent Technologies, Santa Clara, CA, USA) equipped with a HP-5MS capillary column (60.0 m × 250 μm × 0.25 μm, Agilent, Santa Clara, CA, USA). Helium was used as the carrier gas (linear velocity of 1.8 mL min^−1^) at a flow rate of 1.5 mL min^−1^. The temperature program was isothermal at 50 °C for 2 min, and increased to 100 °C at 5 °C min^−1^ and held for 2 min, increasing to 150 °C at 4 °C min^−1^ and held for 1 min, increasing to 180 °C at 4 °C min^−1^, held for 2 min, and finally, the temperature was raised to 250 °C at 5 °C min^−1^ and held for 5 min. The mass spectrometer was operated in the electron impact mode with the electron energy set at 70 eV. The ion source temperature was set at 230 °C, with a mass range of 33–550 amu (*m*/*z*). The identification of the volatile substances was carried out by comparing their retention times and mass spectra data with those from the Wiley and NIST digital libraries.

### 2.7. Data Analysis

All measurements were performed in triplicate, and all results were expressed as mean ± standard errors. One-way ANOVA and Duncan’s multiple range test (IBM SPSS Statistics R24, IBM Corporation, Armonk, NY, USA) was conducted to analyze significant differences. A *p* ≤ 0.05 value was considered statistically significant. The principal component analysis and cluster analysis were carried out using R Studio (Version 1.2.5019).

## 3. Results and Discussion

**Figure 1:**
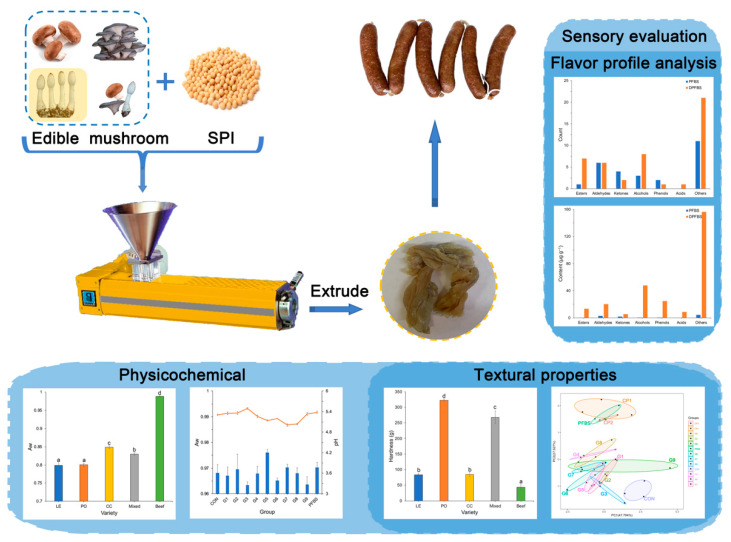
Schematic diagram of the preparation of mushroom-based meat sausage analogue (MMSA).

Data from the Food and Agriculture Organization of the United Nations (FAO) show that more than 100 countries around the world are cultivating mushrooms, with China being responsible for more than 70% of global production [16]. Incorporating mushrooms in meat analogues could offer an opportunity to develop more sustainable and healthier meat-like products. Therefore, SPI and different edible mushrooms (*Lentinus edodes*, *Pleurotus ostreatus*, *Coprinus comatus* and a mixture of equal proportions) were mixed and converted into edible mushroom-based meat analogues using extrusion technology. To explore the applications of this meat analogue, it was further processed to develop value-added products. Figure 1 shows that the changes in formulation and processing greatly affected the organoleptic characteristics and consumer acceptability in terms of the texture, sensory and flavor profiles.

### 3.1. Manufacturing of Edible Mushroom-Based Meat Analogue

**Figure 2:**
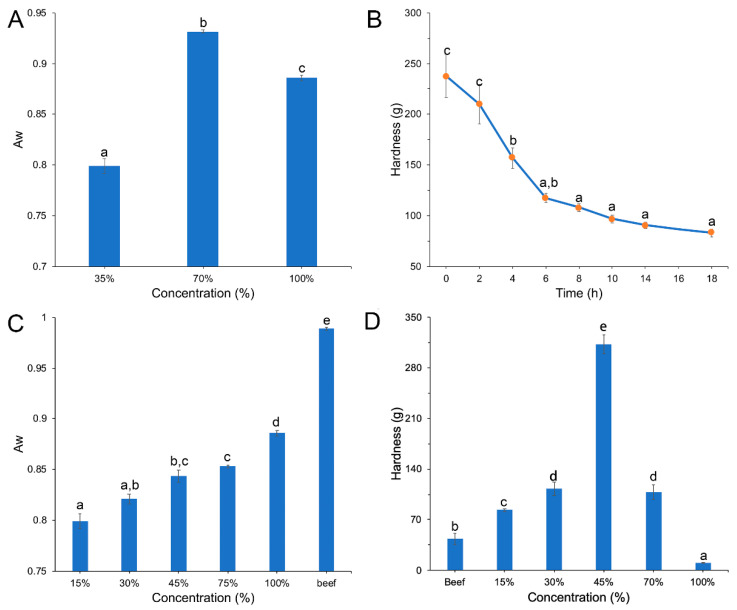
(**A**) The *a_w_* of mushroom-based meat analogues with water contents from 35% to 100%; (**B**) The hardness of mushroom-based meat analogues with different rehydration times; (**C**) The *a_w_* and (**D**) hardness of mushroom-based meat analogues with contents of edible mushroom from 15% to 100% compared with beef.

The edible mushroom-based meat analogue was successfully produced using thermos-extrusion. When squeezed out of the machine, the extrudate was expanded and formed a characteristic meat-like fiber structure because the water in the ingredients was evaporated promptly when released from the high temperature and high pressure. The water content is crucial to the structure of extrudates because the mobility of proteins, cross-linking and water absorption are all affected by moisture [17]. In the present study, water contents of 35%, 70% and 100% were used in the ingredients and the *a_w_* of the extrudate was evaluated. Figure 2A shows that only at a water content of 35%, was the *a_w_* of the extrudate under 0.85, considered a relatively ideal value because it does not support microbial growth. Therefore, 35% was chosen as suitable water content for further experiments. An extrudate with a water content under 45% should be rehydrated before further application [18]. The time for rehydration in the present study was optimized based on the hardness of the extruded edible mushroom-based meat analogue (Figure 2B). The hardness gradually decreased as the extrudate was soaked in water until it became stable after 8 h, so this was set as the appropriate time of rehydration for further experiments.

The edible mushroom content significantly affected the flavor and textural properties, greatly influencing the final characteristics and the added value of the products. In the present study, between 15% and 100% mushrooms were in the ingredients for producing the meat analogues. All the extrudates emerged from the dye successfully, and the *a_w_* was increased with the amounts of mushroom (Figure 2C). While the content of mushroom was more than 45%, the *a_w_* was more than 0.85, a condition facilitating the growth of microorganisms. After rehydration, the meat analogue with 100% added edible mushrooms was dispersed in water with the solids presenting as a mud of almost negligible hardness, thus restricting further processing. The discrepancies in structure and textural characteristics between meat analogues with different levels of added edible mushroom and beef were evaluated using a texture profile analysis (TPA). Compared with beef, the meat analogue was the most similar in the hardness value when mushroom addition was 15% (Figure 2D), with the fracture properties exhibiting a similar trend (Figure S1), while there were no differences in springiness and viscosity between meat analogues (Figures S2 and S3). With regard to its shelf-life and textural properties, an addition of 15% was a suitable proportion of mushroom in the meat analogue.

To meet consumer expectations, meat analogues have been developed to simulate the appearance, texture, flavor and taste characteristics of animal meat products. Selecting an edible mushroom for use in formulating meat analogues can also greatly influence the organoleptic characteristics of the final products [19]. Thus, *Lentinus edodes* (LE), *Pleurotus ostreatus* (PO), *Coprinus comatus* (CC) in a mixture of equal proportions were used for the meat analogues based on the suitable water content (35%) and mushroom amount (15%). The *a_w_* of extrudate from CC was the greatest of the extrudates but all were less than 0.85 (Table 2), indicating they were suitable for product storage and prolonged shelf life to a certain extent. The use of different species of mushrooms in the ingredients significantly influenced the texture of the meat analogues (Table 2). The hardness of the meat analogue from PO was remarkably high and the mixture containing PO was also significantly high, possibly because of its rich content of dietary fiber [20]. While the experimental results showed that softer products were formed when the LE and CC mushrooms were used, their hardness values were closest to those of beef. Nevertheless, the springiness of the meat analogues and different species of mushroom was similar.

**Figure 3:**
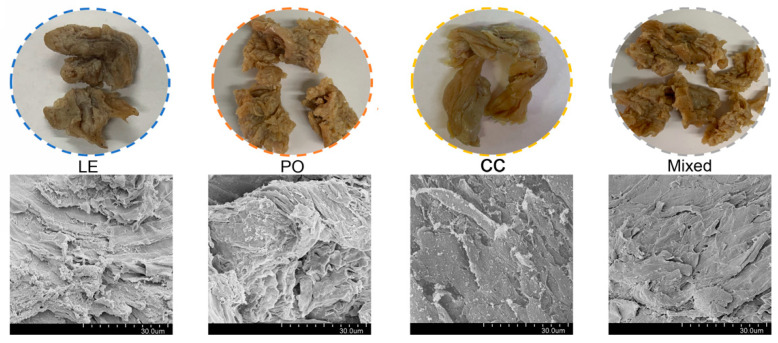
The texture information obtained from photographs of meat analogues containing edible mushroom from LE (blue), PO (orange), CC (yellow) and the mushroom mixture (grey) and corresponding inner microstructure information obtained from SEM.

The appearance data of extrudates made from the different types of edible mushrooms are shown in Figure 3. It was obvious that the extrudates from CC and PO exhibited a characteristic fibrous structure as well as a satisfying luminosity, while extrudates from LE and the mixture containing LE were matt with several dark spots. The inner microstructures of the extrudates made from different types of mushrooms were observed using SEM, which displayed the layered structure of extrudates to some degree. Compared with extrudates made from PO and the mixture, the extrudates with LE and CC appeared more homogeneous, with an orderly fibrous structure. In particular, extrudates from PO exhibited a more compact and helical fibrous structure, which might be the reason for its highest hardness level, and indicated that PO and the mixture were not suitable for protein texturization in this processing treatment. Storage conditions, appearance and texture attributes are well-known to affect consumers’ acceptability and preference of food products [6]. Therefore, the CC edible mushroom was used for formulating the meat analogue.

### 3.2. Manufacturing the Mushroom-Based Meat Sausage Analogue

**Figure 4:**
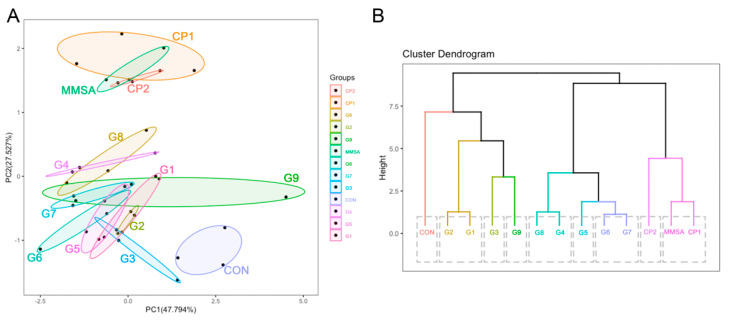
(**A**) Principal component analysis plot and (**B**) cluster analysis of mushroom-based meat sausage analogues comparing different formulations and commercial products.

Egg white powder is commonly used in sausage production because it can improve texture by retaining moisture and stabilizing the product, without adding any unpleasant flavors. Meat flavor powder is a well-known flavor enhancer, but its excessive use can result in the scent of spices becoming unacceptable. Red yeast rice was produced by *Monascus purpureus*, which has been reported to reduce cholesterol levels, affect texture properties, give sausages their characteristic meat-like appearance, improve color stability, enhance flavor and stimulate the appetite [21,22]. Plant oils also act as an excellent lipid ingredient and can be included in a formulation to enhance the tenderness, mouthfeel, juiciness and flavor release of the meat product [6]. Therefore, the contents of egg white powder, red yeast rice, and oil were selected as key factors for optimization. To obtain a holistic view of the acceptability and preference of the products, the sensory attributes of the different sausage analogue samples were assessed comprehensively by sensory evaluation. Before steaming, the physicochemical properties, pH and *a_w_*, of the sausage analogues were measured and compared. The *a_w_* and pH of the sausages ranged from 0.96 to 0.98 and from 5.0 to 5.8, respectively (Figure S4), values slightly lower than those of traditionally made animal meat sausages but might improve shelf-life [23]. The color of different groups was measured with a colorimeter. The results in Table S1 shown the strong relationship between the lightness and redness values with the amount of red yeast rice. When red yeast rice was added in high levels, the color value of sausage analogues exhibited a lower level. The sensory evaluation focused on color, flavor, mouthfeel and structural condition, which assessed the acceptability of sausage analogue with different formulations more directly (Table S2). Based on the scores of different treatment groups from 32 panelists, the most significant factor is red yeast rice and followed by egg white powder, oil, and meat flavor powder. The optimized formulation of the sausage analogue (MMSA, Figure S5) was as follows: red yeast rice at level II (0.02%), egg white powder at level III (5%), oil at level II (19%), and meat flavor powder at level I (0.1%). As well as sensory evaluation, texture profile is one of the most important properties of sausage products from both animal and plant sources. A principal component analysis (PCA), which is a multivariate statistical method, can highlight differences between the samples [24]. The PCA plot (Figure 4A) reflected statistically significant differences in the core information to allow a comparison of the textural characteristic of sausage samples from the control, treatments (G1–9), and optimal (MMSA) groups on the main component plane. Based on the analysis of hardness, springiness, adhesiveness and fracture properties, the data were clustered into the following three groups: MMSA and the commercial products (CP1 and CP2) overlapped and interwove, indicating that they cannot be completely discriminated and exhibited similar textural characteristics. Meanwhile, they were separated from the interlaced treatment groups and the control group thoroughly, which displayed a significant discrepancy in texture profile. The cluster dendrogram (Figure 4B) further shows the hierarchical relationship between the sausage samples more directly. The cook yield test was carried out and the results were shown in Table S3. After being steamed, the yields were between 101.22% to 106.89%. The increased weight of all the sausage analogues was attributed to the vapor they absorbed. Overall, the evaluation showed that MMSA might be an ideal and feasible formulation for producing sausages made from plants and edible mushrooms to replace those made using animal resources. While it is might not be suitable for vegetarians who do not eat any meat products and consume eggs due to the existence of egg powder and meat flavor powder.

### 3.3. The Flavor Profile and Comparison of Meat Sausage Analogue

Flavor is also considered an important sensory characteristic of meat products and largely determines the purchase intent of the consumer. Proteins have little flavor, but many studies have reported their ability to bind and retain flavor compounds [25,26]. Naturally fermented sausages, such as the Harbin dry sausage, are well known and very popular among the people of northeast China because of their mouthfeel, distinctive flavor and short natural fermentation period (15 days). To explore a greater range of applications, a dry-fermented sausage based on MMSA was also developed by hanging sausages and drying them at 25 ± 2 °C for 15 days (30% relative humidity). To portray the flavor profile, the volatile substances in both MMSA and the dried MMSA (DMMSA) were extracted and analyzed by HS-SPME combined with GC-MS.

**Figure 5:**
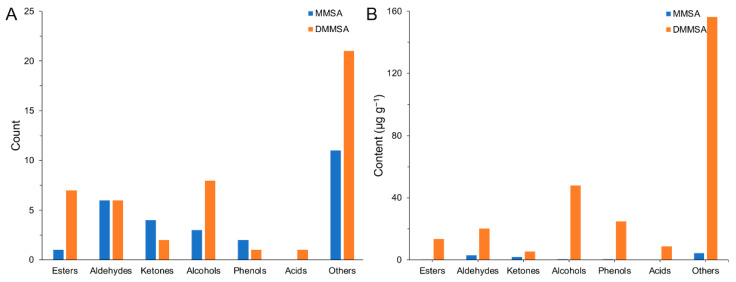
(**A**) The number of different types of volatiles in MMSA and DMMSA; (**B**) The content of different types of in MMSA and DMMSA.

A total of 64 volatile compounds from the MMSA and DMMSA samples were identified and quantified, and they were grouped as esters, aldehydes, ketones, phenols, acids and other compounds. To view the differences of volatile substances in the sausages comprehensively, the number and contents of different chemical classes are exhibited in Figure 5. Generally speaking, the number of different classes in DMMSA was greater than that in MMSA except for ketones and phenols. The contents of volatile compounds from the different classes showed a great difference. 10.372 µg·g^−1^ and 276.603 µg·g^−1^ volatile compounds were detected in MMSA and DMMSA, respectively. The content of each class in DMMSA was much higher than that in MMSA, because more volatile compounds had been generated during the dry fermentation processing. The complexities of lipid oxidation and bacterial metabolism are responsible for the abundance of volatile flavor substances that contributed to the aroma profile.

The number of volatile substances identified in the MMSA and DMMSA samples was 27 and 45, respectively (Table 3). Hexanal, described as the fat odor, was the most abundant of all the volatile substances in MMSA at 1.931 µg g^−1^, and is the most typical of the aldehydes generated by lipid autoxidation [27]. Other aldehydes with certain off-flavors, such as heptanal, 2-heptenal (fat), octanal (fat), 2-octenal (green), 2-nonenal (fat), 2,4-nonadienal, 2-decenal (tallow), 2,4-decadienal (fat) and 9-octadecenal, are also associated with lipid oxidation, and they were not detected in the MMSA sample, except for 1-nonanal with its green odor found at a low level, which might cause deterioration in meat quality and an unpleasant taste [28,29]. Alcohols accounted for 17.26% of all the volatile compounds in DMMSA, with linalool (floral, sweet) being the most abundant alcohol at 17.138 µg·g^−1^. The content of linalool and limonene (50.853 µg·g^−1^) in DMMSA increased significantly compared with the unfermented MMSA, as reported in a previous study on dry-fermented sausages. The contents of other compounds originating from spices such as alpha-terpineol, cinnamaldehyde, phellandrene, alpha-terpineol, alpha-curcumene, bisabolene and alpha-pinene were also at a high level in DMMSA and may contribute to oxidation and the microbial enzyme metabolism [30]. The results for DMMSA agreed with Wen’s, work which reported that the contents of volatile compounds from spices were higher in air-dried sausages than in other types [31]. Limonene was the second most abundant volatile compound detected in MMSA, mainly originating from the spices [32]. The odor of 2-heptanone, with its spicy, blue cheese odor and high threshold, was present at 0.943 µg·g^−1^ in MMSA, which might result in an adverse contribution to the aroma profile. Furthermore, 4-Allylanisole, which was regarded as an anisic, sweet, spicy odor, and the only ether detected in the sausage samples, was the second most abundant volatile in DMMSA at 24.840 µg·g^−1^. DMMSA was richer in esters and aldehydes which have the most important role in product flavor because of their low threshold [33]. Of the aldehydes, benzaldehyde is associated with almond flavor derived from the bacterial catabolism of amino acids and has been detected at high levels during the air-drying of fermented sausages [28,34]. Linalyl acetate, with a green herbal aroma, accumulated at high levels in DMMSA (4.215 µg·g^−1^). The only acid in the sausage samples was acetic acid, detected in DMMSA at 8.626 µg·g^−1^, probably originating from the catabolism of carbohydrate by *P. pentosaceus* carbohydrate catabolism [35].

The composition of meat is affected by the animal species, breed, feeding regime and other factors [36]. However, proteins and lipids are the main substrates for the chemical reactions and microbial metabolism responsible for forming flavors in animal-based sausages [37]. As the meat analogues were prepared from protein-rich ingredients, plant-based sausage analogues might be involved in a similar pathway for forming aromas in their animal meat counterpart [38,39]. Additionally, 1-Hexanol, acetic acid, hexanal, nonanal, 3-hydroxy-2-butanone, hexanoic acid, 2,3-butanedione, 2-methyl butanoate, β-myrcene, α-terpinene, terpinolene and linalool, have often been detected in fermented sausages inoculated with a starter culture [27]. In animal-based dry-fermented sausages which undergo spontaneous fermentation, hexanal, octanal, nonanal, 3-hydroxy-2-butanone, 1-octen-3-ol, ethyl acetate and ethyl hexanoate are essential for forming the overall flavor profile. In the present study, 1-hexanol, acetic acid, nonanal, linalool, α-terpinene and terpinolene were detected, as well as flavor compounds uniquely found in traditional dry sausages from Northeast China, such as 4-allylanisole, cineole and linalool which were also found in DMMSA at high levels [40]. Meanwhile, protein has been proved to contribute to the retention of aroma compounds from the addition of substances such as flavorings and spices [18]. These results indicated that the profile of the sausage analogue could be close to that of real meat sausages, in both flavor and taste through adsorbing and maintaining the spices.

## 4. Conclusions

To mimic the quality characteristics of animal-based sausage, the meat analogue was successfully prepared and used in formulations of sausages. The formulation of the mushroom-based meat analogue was investigated and evaluated by incorporating different species and amounts of mushroom with SPI. The results of physicochemical, textural profile, appearance and inner microstructure analysis showed that meat analogue prepared from CC (15% addition) and SPI, with a water content of 35% was mostly close to beef, which implied the edible mushroom was successfully applied to produce a meat substitute. Moreover, the formulations and processing treatment of sausages analogues greatly influenced the physicochemical, textural, sensory and volatile compounds of MMSA. Furthermore, the volatile compounds from MMSA and DMMSA exhibited the outstanding distinction of different processes, which indicated that naturally fermented non-animal meat resources could provide a great variety of aromas. Ultimately, the present study developed a novel mushroom-based meat sausage analogue with a satisfactory level of consumer acceptability, which could contribute towards reducing the consumption of animal-based meat and provide more opportunities for developing and selling non-animal sourced products.

## Figures and Tables

**Table 1:** Key factorial design for mushroom-based meat sausage analogues formulation.

Treatments	Egg White Powder (%)	Meat Flavour Powder (%)	Oil (%)	Red Yeast Rice (%)
Control	0	0	0	0
G1	3	0.1	17	0.01
G2	3	0.2	19	0.02
G3	3	0.3	21	0.03
G4	4	0.1	19	0.03
G5	4	0.2	21	0.01
G6	4	0.3	17	0.02
G7	5	0.1	21	0.02
G8	5	0.2	17	0.03
G9	5	0.3	19	0.01
MMSA	3	0.1	19	0.02

**Table 2:** The *a_w_*, hardness, springiness, fracture property and viscous force of MMSA with different species of edible mushroom and compared with beef.

Species	*a_w_*	Hardness (g)	Springiness (mm)	Fracture Property (g)	Viscous Force (g)
LE	0.80 ± 0.01 ^a^	83.17 ± 3.75 ^a^	61.54 ± 0.29 ^a^	66.50 ± 3.75 ^bc^	3.17 ± 0.29 ^a^
PO	0.80 ± 0.00 ^a^	322.00 ± 4.33 ^a^	59.90 ± 2.30 ^a^	124.85 ± 2.03 ^d^	3.00 ± 0.50 ^a^
CC	0.85 ± 0.00 ^b^	84.17 ± 6.11 ^c^	61.31 ± 0.30 ^a^	55.53 ± 3.71 ^b^	3.33 ± 0.29 ^a^
Mixed	0.83 ± 0.00 ^b^	268.00 ± 20.39 ^b^	60.49 ± 1.25 ^a^	84.75 ± 3.71 ^c^	2.67 ± 0.29 ^a^
Beef	0.99 ± 0.00 ^c^	43.17 ± 7.77 ^d^	61.95 ± 0.69 ^a^	19.77 ± 2.40 ^a^	3.33 ± 0.29 ^a^

^a–d^ Means within the same row with s differ significantly among the treatments (*p* ≤ 0.05).

**Table 3:** Volatile compounds extracted from MMSA (µg g^−1^) and DMMSA and analyzed using HS-SPME combined with GC-MS.

No.	Volatile Compounds	RI (s)	CAS3	Content (µg·g^−1^)	
1	Acetic acid	4.4785	64-19-7		8.626
2	Isovaleraldehyde	4.978	590-86-3	0.145	2.01
3	Heptane	5.5244	142-82-5		1.892
4	2-Ethylfuran	5.5833	3208-16-0	0.628	
5	trans-2-Pentene	6.0708	646-04-8		1.948
6	Toluene	6.7994	108-88-3		7.316
7	2,3-Butanediol	7.0226	513-85-9		1.954
8	Hexanal	7.393	66-25-1	1.931	
9	5-Hexenenitrile	8.9322	5048-19-1		3.006
10	1-Hexanol	9.07035	111-27-3	0.207	6.493
11	2-Heptanone	9.6962	110-43-0	0.943	
12	1,3,5,7-Cyclooctatetraene	9.8372	629-20-9	1.084	
13	Heptaldehyde	10.0076	111-71-7	0.307	
14	2,5-Dimethyl pyrazine	10.32775	123-32-0	0.345	2.031
15	Methyl hexanoate	10.6596	106-70-7		1.615
16	alpha-Pinene	11.11505	80-56-8	0.091	2.413
17	Benzaldehyde	11.9112	100-52-7	0.29	5.803
18	1-Octen-3-ol	12.3637	3391-86-4	0.17	
19	beta-Pinene	12.5281	127-91-3		2.427
20	3-Octanone	12.6399	106-68-3	0.148	
21	2(5H)-Furanone	13.2098	497-23-4	0.729	
22	alpha-Phellandrene	13.4623	99-83-2		2.310
23	3-Carene	13.6914	13466-78-9		9.185
24	alpha-Terpinene	13.9029	99-86-5		1.395
25	p-Cymene	14.1967	99-87-6		6.791
26	Limonene	14.3613	138-86-3	1.553	50.853
27	1,8-Cineole	14.4905	470-82-6		10.458
28	gamma-Terpinene	15.4834	99-85-4		2.684
29	4-Thujanol	15.8184	546-79-2		2.229
30	2-Ethyl-3,5-dimethylpyrazine	16.1711	55031-15-7	0.199	
31	1-Adamantanol	16.3004	768-95-6	0.153	
32	2-Nonanone	16.6059	821-55-6	0.057	
33	(−)-Fenchone	16.6938	7787-20-4		4.263
34	Linalool	16.9465	78-70-6		17.138
35	1-Nonanal	17.0964	124-19-6	0.269	2.174
36	Maltol	17.4813	118-71-8	0.392	
37	Caprylic acid methyl ester	17.8337	111-11-5		1.357
38	Terpinolene	20.1134	586-62-9		1.944
39	(−)-alpha-Terpineol	20.5834	10482-56-1		1.897
40	Dodecane	20.7364	112-40-3	0.078	
41	4-Allylanisole	20.8302	140-67-0		24.840
42	Decyl aldehyde	20.9714	112-31-2	0.059	
43	Cuminaldehyde	22.4578	122-03-2		0.628
44	L(−)-Carvone	22.5635	6485-40-1		1.055
45	Linalyl acetate	22.8162	115-95-7		4.215
46	p-Anisaldehyde	22.9396	123-11-5		5.507
47	Cinnamaldehyde	23.5447	104-55-2		4.093
48	Tridecane	24.391	629-50-5	0.176	
49	Cinnamyl alcohol	24.7375	104-54-1		5.638
50	Terpinen-4-ol	25.9831	562-74-3		0.876
51	Terpinyl propionate	26.2769	80-27-3		1.378
52	2,6,11-Trimethyldodecane	27.0468	31295-56-4	0.036	
53	Tetradecane	27.8224	629-59-4	0.155	
54	beta-Caryophyllene	28.9209	87-44-5		24.365
55	alpha-Caryophyllene	30.0255	6753-98-6		1.622
56	alpha-Curcumene	30.7188	644-30-4		5.028
57	alpha-Zingiberene	31.089	495-60-3		2.916
58	beta-Bisabolene	31.5296	29837-09-0		1.883
59	Butylated hydroxytoluene	31.6473	128-37-0	0.032	
60	1-Hexadecene	34.121	629-73-2	0.056	
61	Homosalate	43.4571	118-56-9		0.479
62	Methyl palmitate	44.13575	112-39-0	0.139	1.025
63	Dibutyl phthalate	45.1492	84-74-2		3.431
64	N-Eicosane	45.8954	112-95-8		0.572

## Supplementary Materials

The following are available online at https://www.mdpi.com/article/10.3390/foods11010052/s1, Figure S1: Fracture properties of meat analogues, Figure S2: Springiness of meat analogues, Figure S3: Viscosity of meat analogues, Figure S4: The *a_w_* and pH of uncooked meat sausage analogues, Figure S5: The picture of MMSA taken by a camera under sunlight, Table S1: Changes in L*, a*, b* values of sausage analogues, Table S2: Sensory analysis of key factors at different level, Table S3: Cook yield of sausage analogues.
