# Supplementary material for: Textural, Sensory and Volatile Compounds Analyses in Formulations of Sausages Analogue Elaborated with Edible Mushrooms and Soy Protein Isolate as Meat Substitute
Source: Foods. 2021 Dec 27;11(1):52. doi: 10.3390/foods11010052 (PMC8750815; doi:10.3390/foods11010052)
Supplement: Supplementary file 1 [file foods-11-00052-s001.zip › foods-1515433-supplementary.pdf]

Textural, sensory and volatile compounds analyses in formula-tions of sausages analogue elaborated with edible mushrooms and soy protein isolate as meat substitute

Xinyue Yuan, Wei Jiang, Dianwei Zhang, Huilin Liu*, and Baoguo Sun

Beijing Advanced Innovation Center for Food Nutrition and Human Health, Beijing Engineering and Technology Research Center of Food Additives, Beijing Technology and Business University, 11 Fucheng Road, Beijing, 100048, China.

* Corresponding author:

Corresponding author: Huilin Liu

Tel: (86 10) 68984857;

Fax: (86 10) 68984003;

Email: liuhuilin@btbu.edu.cn (Huilin Liu)

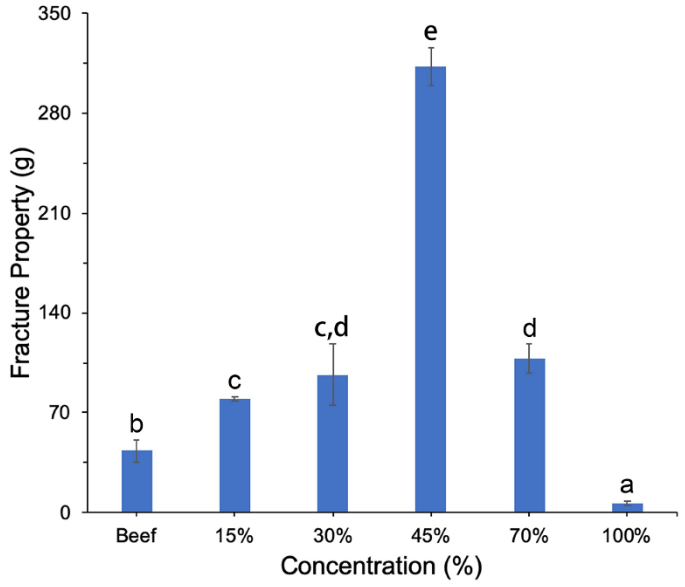

Figure S1 Fracture properties of meat analogues

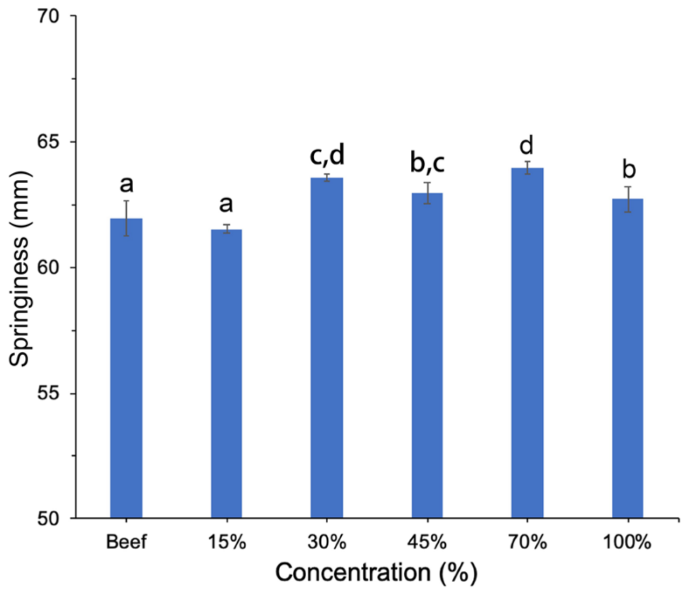

Figure S2 Springiness of meat analogues

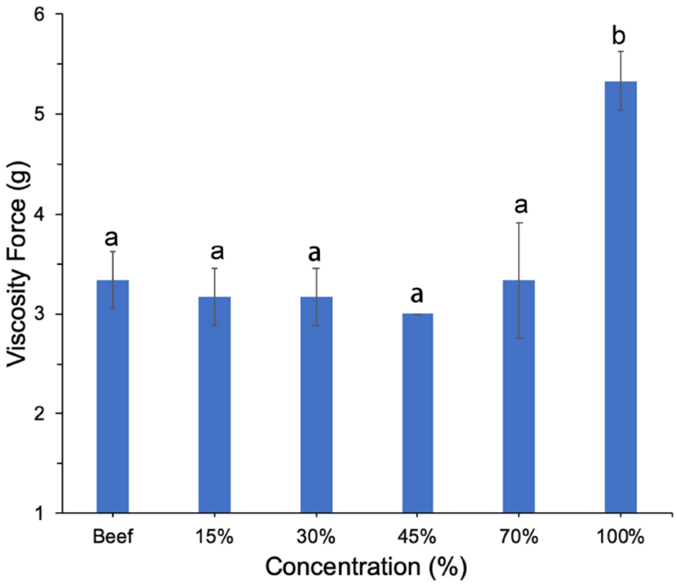

Figure S3 Viscosity of meat analogues

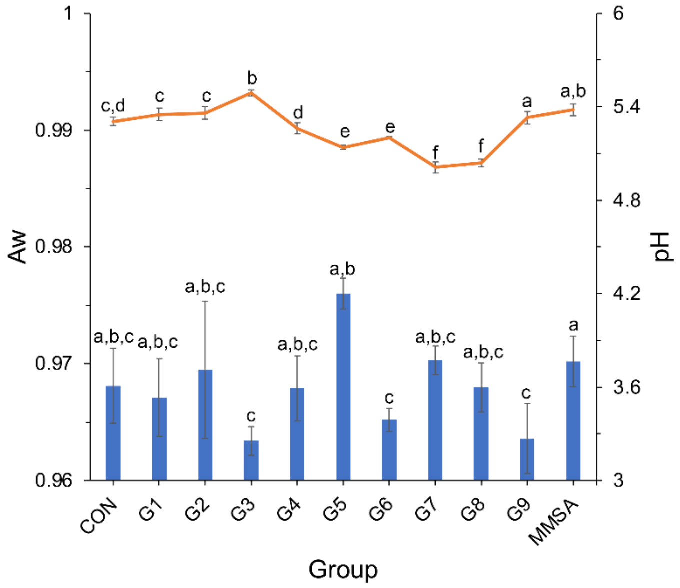

Figure S4 The a_w and pH of uncookd meat sausage analogues

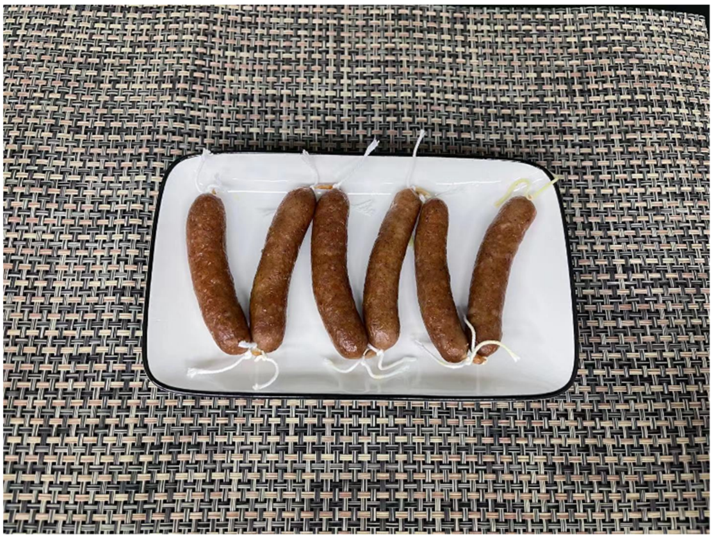

Figure S5 The picture of MMSA taken by a camera under sunlight.

Table S1. Changes in L*, a*, b* values of sausage analogues.

**Treatments**	**L***	**a***	**b***
Control	41.66 ± 0.31	6.78 ± 0.06	16.44 ± 0.17
G1	38.76 ± 0.30	8.72 ± 0.16	13.89 ± 0.34
G2	38.94 ± 0.51	10.83 ± 0.21	13.48 ± 0.47
G3	39.29 ± 0.34	13.89 ± 0.23	17.08 ± 0.26
G4	33.46 ± 0.58	12.24 ± 0.47	16.02 ± 0.55
G5	39.60 ± 0.30	11.69 ± 0.32	15.76 ± 0.17
G6	38.90 ± 0.31	11.25 ± 0.30	14.40 ± 0.35
G7	41.50 ± 0.09	11.71 ± 0.08	17.36 ± 0.15
G8	33.62 ± 0.52	9.73 ± 0.11	14.09 ± 0.16
G9	43.89 ± 0.35	12.50 ± 0.22	19.59 ± 0.08
MMSA	36.53 ± 0.04	20.50 ± 0.06	16.59 ± 0.07
Treatments	Pre-cooked weitght (g)	Cooked weight (g)	Cook yield (%)

Table S2 Sensory analysis of key factors at different level.

Treatments	Egg white powder	Meat flavour powder	Oil	Red yeast rice	Scores
Control	0	0	0	0	28.03
1	1 (3%)	1 (0.1%)	1 (17%)	1 (0.01%)	30.97
2	1 (3%)	2 (0.2%)	2 (19%)	2 (0.02%)	32.1
3	1 (3%)	3 (0.3%)	3 (21%)	3 (0.03%)	35.61
4	2 (4%)	1 (0.1%)	2 (19%)	3 (0.03%)	39.55
5	2 (4%)	2 (0.2%)	3 (21%)	1 (0.01%)	29.45
6	2 (4%)	3 (0.3%)	1 (17%)	2 (0.02%)	33.45
7	3 (5%)	1 (0.1%)	3 (21%)	2 (0.02%)	34.97
8	3 (5%)	2 (0.2%)	1 (17%)	3 (0.03%)	37.42
9	3 (5%)	3 (0.3%)	2 (19%)	1 (0.01%)	33.87
I	98.68	105.49	101.84	94.29	
II	102.45	98.97	105.52	100.52	
III	106.26	102.93	98.93	112.58	
K1j	32.89	35.16	33.95	31.43	
K2j	34.15	32.99	35.17	37.97	
K3j	35.42	34.31	32.98	37.53	
R	2.53	2.17	2.19	6.54	

Table S3. Cook yield of sausage analogues.

Treatments	Pre-cooked weitght (g)	Cooked weight (g)	Cook yield (%)
Control	20.45	21.48	105.04
G1	21.08	21.88	103.80
G2	19.50	20.08	102.99
G3	20.11	20.50	101.93
G4	20.02	20.62	103.02
G5	18.67	19.05	102.05
G6	19.94	20.18	101.22
G7	21.85	22.14	101.33
G8	20.70	22.12	106.89
G9	20.47	21.66	105.82
MMSA	20.31	21.02	103.50
